# Sequence and phylogenetic analysis of the mitochondrial genome for a giant clam commensal shrimp *Anchistus australis* (Decapoda: Caridea: Palaemonidae)

**DOI:** 10.1080/23802359.2019.1700839

**Published:** 2019-12-18

**Authors:** Helu Liu

**Affiliations:** Institute of Deep-sea Science and Engineering, Chinese Academy of Sciences, Sanya, China

**Keywords:** *Anchistus australis*, commensal shrimp, Decapoda, tridacnid clams

## Abstract

The shrimp *Anchistus australis* was a giant clam commensal species. Here, we explored in detail its mitochondrial (mt) genome, which was 15,396 bp in length, containing 13 protein-coding genes, 2 ribosomal RNA genes, 21 transfer RNA genes, and a non-coding control region 429 bp in length. The overall mt genome organization of *A. australis* displayed the typical characters for the pancrustacean ground pattern, with exception of translocation of *trnW* and *trnL_1_*, as well as deletion of *trnL_2_*. Phylogenetic analysis confirmed its evolutionary relatedness to other shrimp of family Palaemonidae within Infraorder Caridea.

*Anchistus australis* (Bruce 1977) was a tridacnid clam commensal shrimp, belonging to Decapoda, Caridea, Palaemonidae. This species could be in danger due to decreasing number of its host (Neo and Todd 2012), yet no available report showed that this shrimp could be successfully kept in captivity. On May 2017, a female *A. australis* was obtained from the mantle of giant clam, *Tridacna squamosa*, found at a depth of 0.6 m on coral reefs off Sanya (18°12′48.0″N, 109°28′26.9″E), Hainan Province, China. The whole shirmp was used for DNA extraction. Following DNA extraction, fragments for *cytb*, *cox1*, *nd5* and *lrRNA* were amplified by using primer sets from previous study (Lin et al. 2012), and the rest of the DNA sample (accession number: SY_LH_008) was stored in Deep-sea biology laboratory of Institute of Deep-sea Science and Engineering, Chinese Academy of Sciences. Based on these fragments, long PCR primers were designed to determine the whole mitochondrial (mt) genome. Locations of protein-coding genes (PCGs), ribosomal RNAs (rRNA) and transfer RNAs (tRNA) genes were identified with MITOS (Bernt et al. 2013), tRNAscan-SE (http://lowelab.ucsc.edu/tRNAscan-SE/) and ORF Finder (https://www.ncbi.nlm.nih.gov/orffinder/). The mt genome was deposited in the GenBank under accession numbers MN412556.

The complete mt sequence of *A. australis* was 15,396 bp in length, with 68.4% of A + T content. It contained 13 PCGs, 2 rRNA genes, 21 tRNA genes, and 1 non-coding control region 429 bp in length. As in other Decapods (Lin et al. 2012), nine PCGs (*cox1*, *cox2*, *cox3*, *cytb*, *nd2*, *nd3*, *nd6*, *atp6*, *atp8*) were encoded on the H-strand, while other four PCGs (*nd1*, *nd4*, *nd4L*, *nd5*) were encoded on the L-strand. Compared to the pancrustacean ground pattern, overall gene arrange in this genome had no significant change. *trnL_1_*, located between *nad1* and *lrRNA* in other pancrustacea, was found inside the *lrRNA* in *A. australis*. *trnaW* moved downstream to *trnaY.* Additionally, no *trnaL_2_* could be detected. Phylogenetic analysis showed NJ and ML method produce identical tree topologies with congruent support (Figure 1). *Anchistus australis* grouped well into family Palaemonidae, within infraorder Caridea, a monophyletic group with high support (97/94) both in NJ and ML tree.

**Figure 1.**
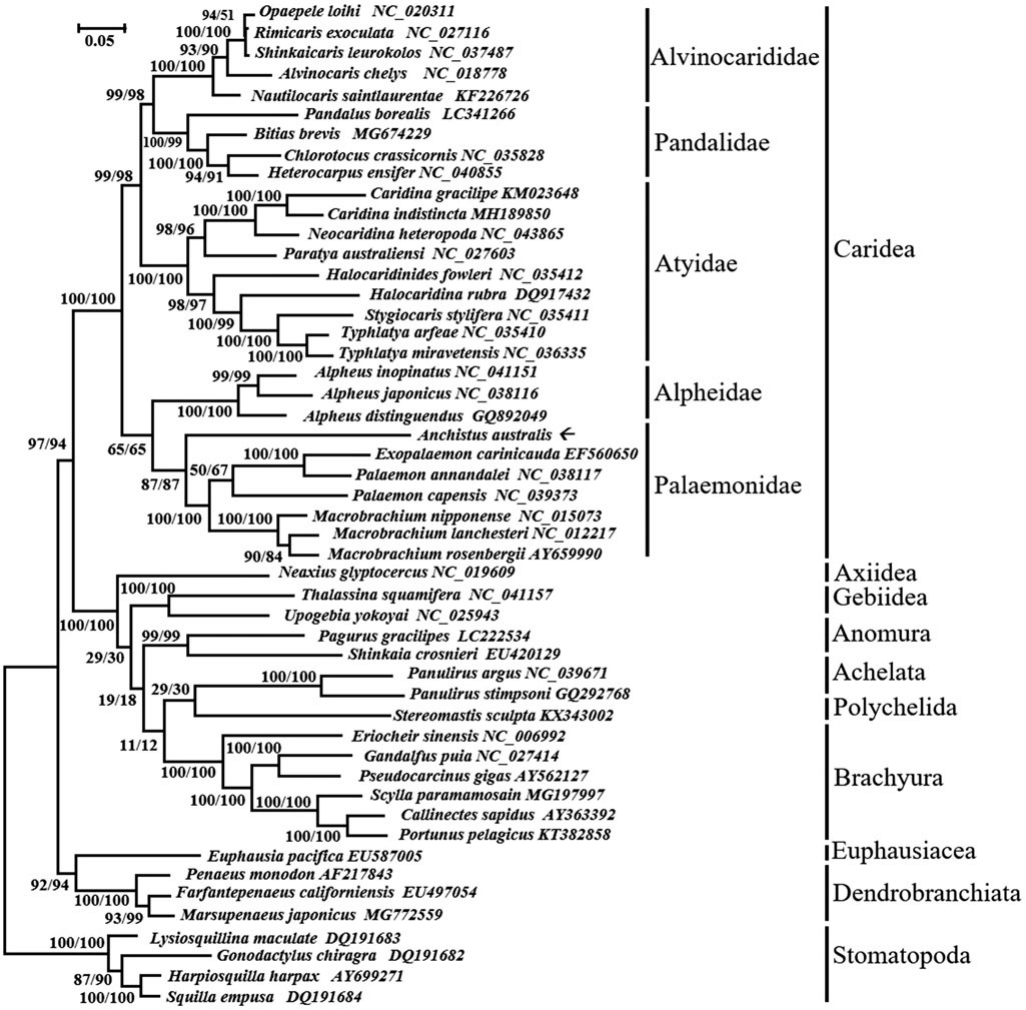
Neighbor-joining (NJ) and maximum-likelihood (ML) phylogeny of *A. australis* and other Decapoda based on the aligned and concatenated protein sequences of 13 protein-coding genes. Both NJ and ML tree were generated with MEGA7 (Kumar et al. 2016) with 1000 bootstrap replicates.
